# An Infant Diagnosed With 22q11.2 Deletion Syndrome Following Frequent Apneic Attacks

**DOI:** 10.7759/cureus.54038

**Published:** 2024-02-11

**Authors:** Yuriko Umeyama, Hiroyuki Tanaka, Hidehito Ota, Yuko Kajiho, Akiko Kinumaki

**Affiliations:** 1 Department of Pediatrics, The University of Tokyo, Tokyo, JPN

**Keywords:** 22q11.2 deletion syndrome, mixed apnea, hypocalcemia, anemia, apneic attack

## Abstract

22q11.2 deletion syndrome (DS) is a microdeletion syndrome that pediatricians may encounter. It has a distinctive presentation and is often diagnosed based on a few characteristic symptoms. However, 22q11.2 DS with apnea as the initial symptom has never been reported. In this report, we describe the case of a one-month-old infant diagnosed with 22q11.2 DS due to apneic attacks. Early diagnosis of 22q11.2 DS is crucial because it enables appropriate intervention.

## Introduction

Chromosome 22q11.2 deletion syndrome (DS) is a prevalent microdeletion syndrome estimated to occur in approximately 1 in 4000 live births. This condition is associated with several clinical manifestations, including a distinctive facial appearance, cardiac malformations, immunodeficiency, hypoparathyroidism, and cleft palate [1]. Limited studies have explored the relationship between 22q11.2 DS and apnea [2]. This study reports a case of an infant who experienced apneic attacks and was subsequently diagnosed with 22q11.2 DS.

## Case presentation

**Figure 1:**
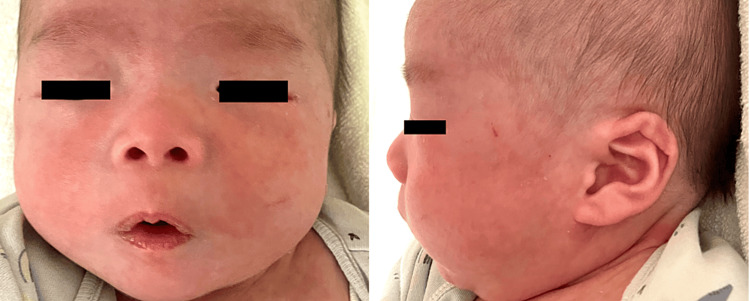
Facial findings on admission Distinct facial attributes were observed, including telecanthus, a depressed nasal bridge, a wide nasal base, a narrow mouth, micrognathia, and low-set and posterior-angulated ears.

**Figure 2:**
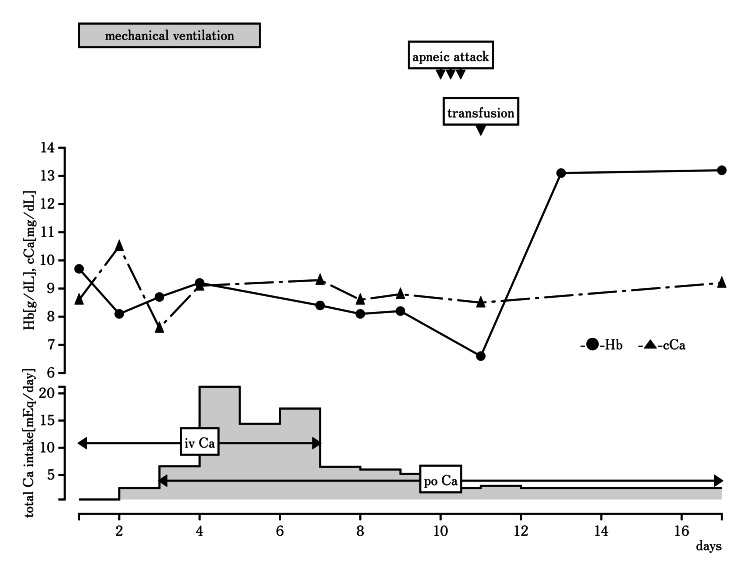
Progress after admission to our hospital Post-hospital clinical course with calcium and Hb levels after hospitalization. At the bottom, the amount of calcium administered to correct calcium levels and the method of administration are noted. After extubation, treatment of anemia and hypocalcemia reduced apneic attacks. cCa, albumin-corrected calcium; iv, intravenous injection; po, per os

The boy was born at 38 weeks of gestation, with a birth weight of 2978 g, to a healthy mother. After discharge, the infant exhibited brief apnea with inspiratory stridor during sleep. On the twenty-ninth day, he was admitted to a local hospital due to recurring apneic attacks with cyanosis. Despite high-flow nasal cannula intervention, apneic attacks worsened without chest or abdominal movements, prompting referral to our hospital. Upon admission, the patient displayed the following vital signs: body temperature, 36.9℃; heart rate, 201/min; respiratory rate, 52/min; blood pressure, 94/57 mmHg; and oxygen saturation (SpO_2_), 87% (2 L/min oxygen administration via nasal cannula). Notably, he exhibited retractive breathing, inspiratory stridor, and generalized reticular cyanosis. Additionally, distinct facial attributes were observed, including telecanthus, a depressed nasal bridge, a wide nasal base, a narrow mouth, micrognathia, and low-set and posterior-angulated ears (Figure 1). Blood tests revealed unremarkable findings, except for albumin-corrected hypocalcemia (8.6 mg/dL) and a slightly elevated serum phosphorus level (7.0 mg/dL). Intubation for airway management and intravenous hypocalcemia correction were started immediately after the initial examination and relying solely on physical findings, mixed apnea was clinically suspected. Several examinations were performed to identify the underlying cause of apneic attacks. However, no abnormalities were detected. A closer examination of the endocrine revealed low intact parathyroid hormone (PTH) levels (16 pg/mL, reference range 10-65 pg/mL) in relation to hypocalcemia. The persistent trends in PTH levels contributed to the diagnosis of primary hypoparathyroidism. After the intervention, he was free without recurrence of apneic attacks or retractive breathing. He was extubated on the fifth day of mechanical ventilation, following a four-hour spontaneous breathing test that confirmed the resolution of apnea. However, apneic attacks without chest or abdominal movements relapsed on the ninth day. Subsequent examination, including laryngoscopy, revealed laryngomalacia, and a blood test indicated anemia with a hemoglobin level of 6.6 g/dL. Anemia gradually worsened after admission (Figure 2). No hemorrhagic lesions were apparent, and the findings of the anemia were attributed to poor hematopoiesis. After positioning instructions and a blood transfusion, apneic attacks resolved, with no further recurrences. Polysomnography revealed an exclusive obstructive apnea pattern, with central apnea notably absent. The results showed that obstructive apnea due to laryngomalacia, which is only addressed by positioning instructions, remained, but central apnea due to hypocalcemia and anemia, which were resolved by the intervention, disappeared. Given the presence of primary hypoparathyroidism, laryngomalacia, and a distinct facial appearance, we suspected 22q11.2 DS. This diagnosis was confirmed using fluorescent in situ hybridization, which specifically detects the region responsible for chromosome 22 long arm q11.2 with the TUPLE1 probe.

## Discussion

In this case, apneic attacks were thought to be mixed-obstructive due to laryngomalacia and central due to hypocalcemia and anemia -both of which are known complications of 22q11.2 DS [3]. Anemia was also contributed to by frequent blood draws and the physiological effects of early infancy. The mechanisms through which hypocalcemia and anemia cause apneic attacks have been suggested to affect the respiratory center and decrease the oxygen delivery capacity respectively [4,5]. Notably, 22q11.2 DS exhibits diverse clinical symptoms, and its diagnosis varies by the patient’s age. While cardiac malformations, immunodeficiency, and hypocalcemia commonly serve as diagnostic triggers during infancy or childhood [1], those lacking these features often remain undiagnosed until later stages of childhood. Despite certain distinctive facial appearances, the extensive phenotypic variation of 22q11.2 DS makes it difficult to diagnose based on facial appearance alone. This case shows a comprehensive set of symptoms - primary hypoparathyroidism, laryngomalacia, and a distinctive facial appearance - that became evident during examination for apneic attacks, enabling an early diagnosis. Few reports have been published on the relationship between 22q11.2 DS and apnea [2]. Furthermore, most reports primarily focus on obstructive apnea, with none addressing apneic attacks as the initial symptom triggering the diagnosis of the syndrome.

## Conclusions

In this study, we report a case of a one-month-old child diagnosed with 22q11.2 DS as a result of an apneic attack. Although there are no reports of 22q11.2 DS being diagnosed as the first manifestation of apneic attack, it is necessary to understand the syndrome and its relationship to apnea in order to closely examine apneic attacks. This study underscores the importance of the early diagnosis of patients with 22q11.2 DS so that appropriate intervention can be provided for laryngomalacia, anemia, and hypocalcemia.

## References

[1] McDonald-McGinn DM, Sullivan KE, Marino B (2015). 22q11.2 deletion syndrome. Nat Rev Dis Primers.

[2] Kennedy WP, Mudd PA, Maguire MA (2014). 22q11.2 Deletion syndrome and obstructive sleep apnea. Int J Pediatr Otorhinolaryngol.

[3] Nissan E, Katz U, Levy-Shraga Y, Frizinsky S, Carmel E, Gothelf D, Somech R (2021). Clinical features in a large cohort of patients with 22q11.2 deletion syndrome. J Pediatr.

[4] Gershanik JJ, Levkoff AH, Duncan R (1972). The association of hypocalcemia and recurrent apnea in premature infants. Am J Obstet Gynecol.

[5] Zagol K, Lake DE, Vergales B (2012). Anemia, apnea of prematurity, and blood transfusions. J Pediatr.

